# Metastable Monolayer Formation through a Connector Structure

**DOI:** 10.1021/acs.jpcc.5c02249

**Published:** 2025-07-09

**Authors:** Simon B. Hollweger, Anna Werkovits, Oliver T. Hofmann

**Affiliations:** Institute of Solid State Physics, 27253 Graz University of Technology, NAWI Graz, Petersgasse 16/II, Graz 8010, Austria

## Abstract

The intentional growth of metastable surface structures of organic molecules adsorbed on inorganic substrates is a challenging task. It is usually unclear which kinetic mechanism leads to the metastable surface polymorph after a deposition experiment. In this work, we computationally investigate a growth procedure that allows to intentionally grow a defined metastable surface structure starting from thermodynamic equilibrium. This procedure is applicable to organic–inorganic interface systems that exhibit a thermodynamically stable *connector* structure that can be exploited to grow the metastable target structure. With specific temperature and pressure changes in the system, a significant yield of the target polymorph can be achieved. We demonstrate this procedure on a simplified microscopic interface system of rectangular molecules adsorbing on a square lattice substrate with kinetic Monte Carlo growth simulations.

## Graphical abstract

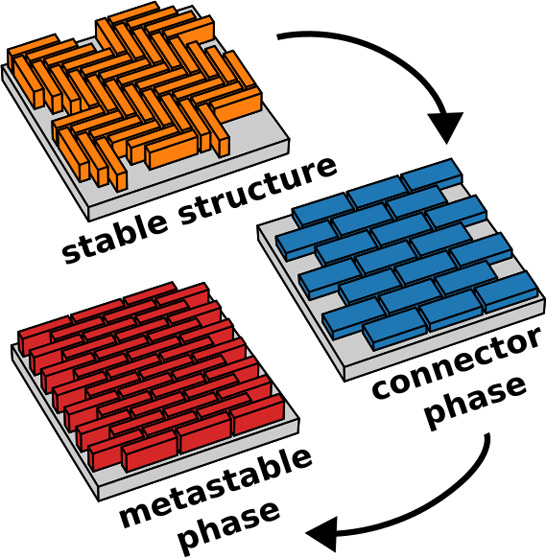

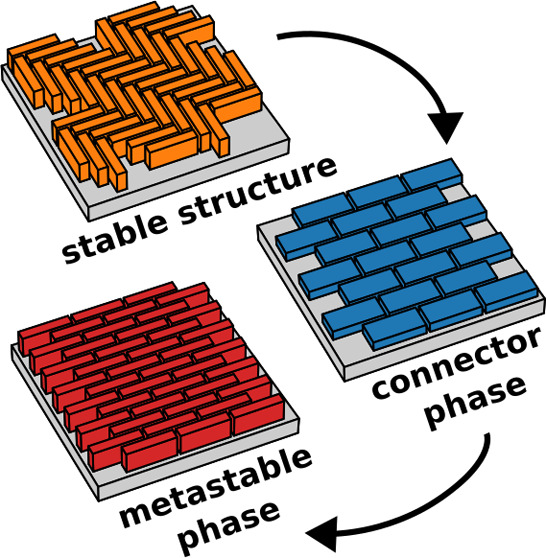

## Introduction

It is well-known that the structure of monolayers of organic molecules adsorbed on metal substrates show pronounced polymorphism depending on their deposition conditions.^1–6^ Controlling which structure the molecules on the surface form by varying certain deposition parameters like temperature and pressure is of great interest, as many interface properties, such as the injection barrier,^7^ charge-carrier mobility,^8^ work function and interface dipole,^9^ depend significantly on the spatial arrangement of the first monolayer of the adsorbates.^10^ However, targeted growth of a certain surface configuration of organic molecules is a highly challenging task, particularly when the desired structure is a metastable polymorph and thus thermodynamically unfavorable.

In this work, we suggest a way to overcome this difficulty by proposing a mechanism for growing a specifically targeted metastable surface structure starting from thermodynamic equilibrium. This procedure is based on two physical/chemical principles: First, in thermodynamic equilibrium the structure that emerges on the surface is the one that minimizes the Gibbs free energy of adsorption per area γ, which is commonly approximated^11,200^ by

$$\gamma_{k}=\frac{1}{A_{k}}\left(E_{k}^{\mathrm{ads}}-\mu \left(T,p\right)N_{k}\right)$$ 1

where $E_{k}^{\mathrm{ads}}$ is the adsorption energy of structure *k*, μ­(*T*, *p*) the chemical potential of the molecular gas reservoir in contact with the surface at temperature *T* and partial pressure *p*, and *N*_*k*_ the number of adsorbed molecules in the unit cell with area *A*_*k*_ (details can be found in the Supporting Information). Due to the linear dependence of γ on μ, it is possible to change the thermodynamically most stable surface structure by a targeted change of the chemical potential of the molecular gas reservoir by the variation of the temperature and pressure in the system. The second principle is Ostwald’s rule of stages.^12^ It states that during crystal formation, phases that are geometrically more similar to the initial phase are formed first, before transforming into the thermodynamically stable structure.

With these two concepts in mind, the conditions that allow systematically growing a metastable surface structure starting from thermodynamic equilibrium can be identified. One key feature of these systems is that they need to show the specific Gibbs free energy curve constellation depicted in Figure 1. It consists of three different surface structures. One of them is the metastable target structure (T) and the other two (S and C) are respective thermodynamically stable polymorphs at certain intervals of the chemical potential μ. The decisive feature of this system is the existence of the connector structure (C). This structure must exhibit a different slope in the Gibbs free energy curve compared to the others and, therefore, intersects them at certain chemical potentials μ. Equation 1 shows that the coverage *N*_*k*_/*A*_*k*_ of the structure determines the slope of the Gibbs curve γ_*k*_, i.e., more densely packed structures exhibit steeper Gibbs curves. To conceptually realize this Gibbs curve constellation the surface structures S and T need to be relatively dense, and the connector phase C more loosely packed. To grow the metastable structure, two steps are now required, as indicated in Figure 1:

1. The first step is to create the thermodynamic stable connector phase C on the surface. This can be achieved by setting the chemical potential of the molecular reservoir to the region where the connector structure C is stable. Since C is more loosely packed than S, its relative stability increases when the temperature is raised, i.e. it becomes thermodynamically stable at elevated temperatures. This facilitates the transition into the thermodynamic equilibrium on the surface experimentally, for example by using techniques such as hot-wall epitaxy.^13^ It is also possible to transform the system into structure C with a postdeposition thermal treatment^6^ where first another thermodynamic stable polymorph S is formed and with a temperature increase and a pressure drop a phase transition to the connector structure C is triggered. We will demonstrate this latter technique in a kinetic Monte Carlo growth simulation later in this work.
2. As soon as the connector structure C has formed, μ is increased to a region where polymorph C becomes unstable. Here, Ostwald’s rule of stages comes into play. The system is likely to prefer a transition to the metastable target polymorph T rather than forming the thermodynamic stable polymorph S, if the connector phase C is geometrically more similar to the target polymorph T than the stable polymorph S.

**Figure 1:**
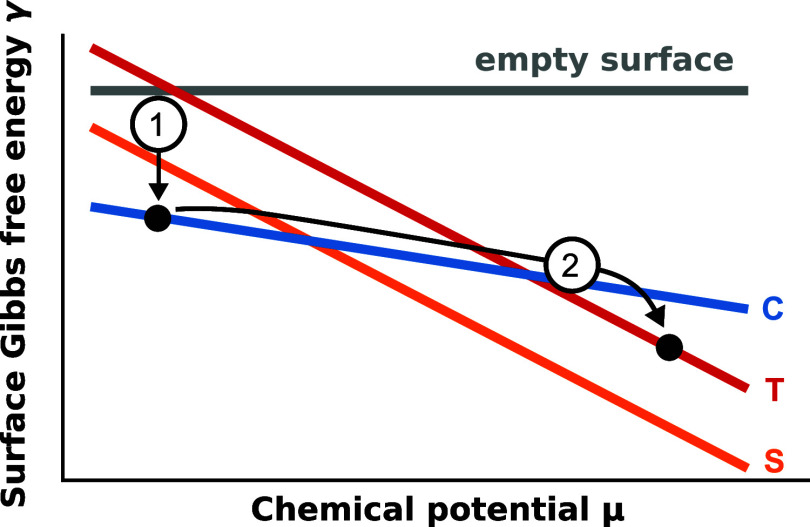
Schematic Gibbs free energy of adsorption per area over gas reservoir chemical potential. The two steps of growing the metastable structure T: (1) The first step is the formation of the connector phase C, (2) the second step is changing the chemical potential to trigger a transformation to the metastable target polymorph T.

We note that the requirement for this procedure is only that (a) at least two different polymorphs are thermodynamically accessible and (b) that the barrier between C and S is not the lowest barrier for phase transformations. Condition (a) is often fulfilled for organic molecules on metal surfaces, as indicated, e.g., by reports about lying-to-standing phase transitions.^14–19^ It is more difficult to make a general statement about (b), but given Ostwald’s rule of stages, it appears at least plausible that also this is often the case.

## Methods

In the simulations, growth is limited to the monolayer regime, as we are mainly interested in designing wetting layers. In our computational experiments the structural evolution of surface adsorbates is modeled in the framework of the *kinetic Monte Carlo (kMC)* method,^20,21^ as implemented in the code *kmos3*.^22,23^ In comparison to *Molecular Dynamics*, *kMC* vastly coarse grains the complex dynamics on atomic scales to a stochastic model that draws and executes so-called *elementary processes*, such as adsorption, desorption and on-surface diffusion/reactions, with a probability proportional to their rate constants. After each step the simulation time is propagated by a Poisson distributed time step. In *kmos3* this is implemented using the *Direct method* (*Variable Step Size method*),^20,21^ i.e. that calculates the time step Δ*t* based on the sum of all rate constants $k_{\mathrm{tot}}$ of processes that are possible at the current step and a uniformly distributed random number r∼U(0,1) :

$$\Delta t=-\frac{\ln(1-r)}{k_{\mathrm{tot}}}$$ 2

However, for time-dependent rates as it is the case in this study, Equation 2 is only a good approximation if the generated time steps Δ*t* are much smaller than the variation time of the rate constants. This is not always the case in the simulation used later in the Results and Discussion section of this study. For a fully closed monolayer where the adsorbed molecules can only perform slow desorption processes and no fast processes like diffusion or reoreintation, the total rate $k_{\mathrm{tot}}$ is relatively small and consequently leads to large time steps Δ*t*. To consider the time-dependence of the rate constants correctly the time steps Δ*t* must be drawn from an inhomogeneous Poisson distribution.^24^ The time steps can be determined by solving

$$\ln(1-r)+\int_{0}^{\Delta t}k_{\mathrm{tot}}(t+\Delta t^{\prime })d\Delta t^{\prime }=0$$ 3

for Δ*t*.^21^ We implemented an algorithm that solves Equation 3 at every kMC step in the used kMC framework *kmos3*.^22^ A detailed description of it can be found in the Supporting Information.

The intermolecular interactions during the growth simulations are treated by the on-the-fly backend of *kmos3*, which evaluates the rate constants during the run. The common time disparity problem in kMC simulations is circumvented by applying the time acceleration algorithm of Dybeck et al.^25,26^

The adsorption rate of molecules on the kMC lattice is derived from classical collision theory of an ideal gas.^27^ The impingement rate of particles on a surface unit cell with area *A*_uc_ at a temperature *T* and pressure *p* is given by

$$k_{\mathrm{tot}}^{\mathrm{ads}}=\frac{pA_{uc}}{\sqrt{2\pi mk_{B}T}}$$ 4

where *m* is the mass of the molecule (we used the mass of an 9–10 anthraquinone molecule *m* = 3.458 · 10^–25^ kg) and *k*_B_ is the Boltzmann constant. This expression only describes the total number of particles per unit time that appear in the unit cell and does not tell anything about which adsorption geometry the molecule assumes after adsorbing. To account for that, it is necessary to estimate the probability *s*_*i*_ that the impinging molecule adsorbs in geometry *i*. For simplicity, we assume that all *N* different adsorption geometries that can occur in a surface unit cell have the same probability *s* = 1/*N*. Essentially, this corresponds to the assumption that it is equally likely for the molecule to impinge on the surface with the flat side or with the edge, and that the sticking coefficient is the same (e.g., unity in a hit-and-stick model). Therefore, the adsorption rate $k_{i}^{\mathrm{ads}}$ for one molecule ending up in geometry *i* is given by

$$k_{i}^{\mathrm{ads}}=sk_{\mathrm{tot}}^{\mathrm{ads}}=s\frac{pA_{uc}}{\sqrt{2\pi mk_{B}T}}$$ 5

The desorption rate constant $k_{\mathrm{des}}$ can be determined by requiring to be consistent with thermodynamics. This requires the adsorption/desorption reaction channel to fulfill detailed balance leading to the expression^27^

$$k_{i}^{\mathrm{des}}\approx k_{i}^{\mathrm{ads}}\exp\left(\frac{E_{i}^{\mathrm{ads}}+E^{\mathrm{int}}-\mu \left(T,p\right)}{k_{B}T}\right)$$ 6

where $E_{i}^{\mathrm{ads}}<0$ is the adsorption energy of an isolated molecule in geometry *i* on the surface, $E^{\mathrm{int}}$ is the interaction energy with the neighboring molecules and μ­(*T*, *p*) is the chemical potential of the molecular gas reservoir. This expression is only an approximation as we estimate the Gibbs free energy of the adsorbed molecule only with its adsorption energy and neglect the vibrational, work and entropic contributions as they are usually small (see Supporting Information).^200,27^

For the on-surface processes such as diffusion, rotation and reorientation processes we used an Arrhenius type rate equation. The rate constant *k*_*ij*_ from adsorption geometry *i* to *j* is then given by

$$k_{ij}=f_{ij}\exp\left(-\frac{\Delta E_{ij}+\Delta \Delta E^{\mathrm{int}}}{k_{B}T}\right)$$ 7

where f_ij_ is the attempt frequency, ΔE_ij_ the corresponding energy barrier and ΔΔE^int^ the correction of the energy barrier resulting from the interaction of neighboring molecules. This correction of the energy barrier is calculated according to the Bronsted-Evans–Polanyi principle^28–33^ and reads

$$\Delta \Delta E^{\mathrm{int}}=\alpha (E_{\mathrm{fin}}^{\mathrm{int}}-E_{\mathrm{ini}}^{\mathrm{int}})$$ 8

where α ∈ [0,1] is a process specific parameter, $E_{\mathrm{ini}/\mathrm{fin}}^{\mathrm{int}}$ is the interaction energy of the initial/final state depending on the specific molecular neighborhood on the surface at the time when the process is executed. A detailed derivation can be found in the Supporting Information.

## Results and Discussion

As a proof of principle, we apply the described procedure of forming a metastable molecular monolayer to a hypothetical microscopic interface model in the framework of kinetic Monte Carlo (kMC).^20,21^ The model consists of rectangular planar molecules adsorbing on a two-dimensional square lattice, representing, e.g., a coinage metal (100) surface. The molecules can adsorb in four distinct adsorption geometries, two lying (face-on) and two standing (long edge-on) orientations, as shown in Figure 2a. Here, we use an adsorption energy (molecule–substrate interaction) of −2.4 eV for the face-on and −2.2 eV for the edge-on configuration, which lies within typical orders of magnitude for the adsorption of conjugated organic molecules on metal surfaces.^34^ The energy barriers between these adsorption geometries are shown in Figure 2c and are inspired by the barriers computed with density functional theory for tetracyanoethylene on Cu(111).^35^ For the face-on geometries, position-dependent interaction energies of ± 55 meV (see Figure 2b) were chosen. Both magnitude- and position-dependence are based on our previous experience with conjugated organic molecules on surfaces.^36^ The interactions between long-edge standing molecules are based on typical interactions of π-conjugated systems.^37^ They penalize a direct overlap of the π-orbitals of the aromatic rings (which would result in Pauli-repulsion) and, therefore, prefer a shifted parallel or a perpendicular orientation between two molecules (Figure 2b). We note that here, the values do not correspond to a specific molecule, but are rather prototypical for the adsorption of conjugated organic molecules on metal surfaces.

**Figure 2:**
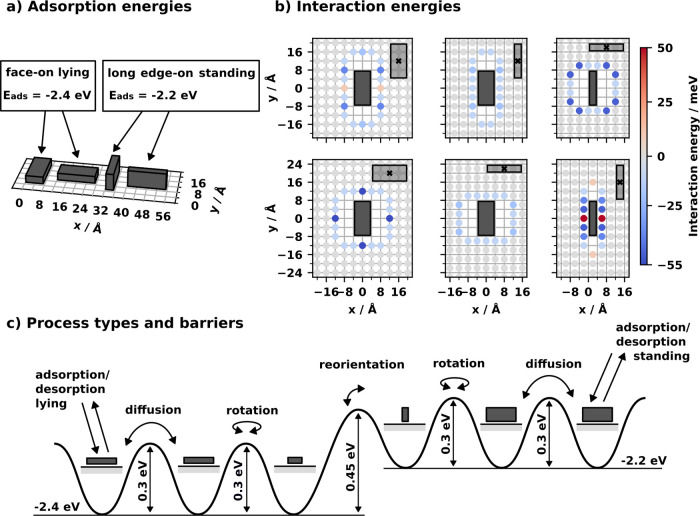
a) Adsorption geometries and energies of the rectangular molecules on a square grid with a lattice constant of 4 Å. b) Pair interaction energies of all combinations of the four adsorption geometries. Each point is a possible position for the geometric center of a neighbor molecule. The color of the points represents the interaction energy between the molecule pair. c) Barriers of the elementary processes (adsorption, desorption, diffusion, rotation and reorientation) between different adsorption geometries. Barriers of that magnitude are representative for conjugated organic molecules and here inspired by the values computed for tetracyanoethylene on Cu(111).^35^ The attempt frequency (prefactor of the Arrhenius equation) of each on-surface process is set to 10^12^ *s* ^–1^, which is typical, e.g. for desorption processes.^39^ We note in passing that the attempt frequency only scales the time required for the phase transitions, but does not affect the physics otherwise.

To connect to thermodynamic conditions, the chemical potential μ of the molecular gas reservoir is approximated by an ideal gas.^27^ To connect μ to pressures and temperatures, typical physical properties of organic molecules were used. Exemplarily, we took the mass, symmetry, and moments of inertia of 9–10 anthraquinone (9,10-dioxoanthracene), but we note that the qualitive findings remain the same for all small organic molecules. The details of the calculation are presented in the Supporting Information.

In our hypothetical model, there are two standing surface structures, a standing brickwall (SBW*) and a standing herringbone (SHB) pattern for the first monolayer, as shown in Figure 3b. The lying molecules can also arrange in a brickwall (LBW) and a herringbone (LHB) pattern. Additionally, we estimate the multilayer (ML) regime of the system by assuming that a molecule adsorbing in the second layer assumes an adsorption energy of –1 eV, which is a typical value for small organic molecules.^38^ This leads to the dotted area in Figure 3a.

**Figure 3:**
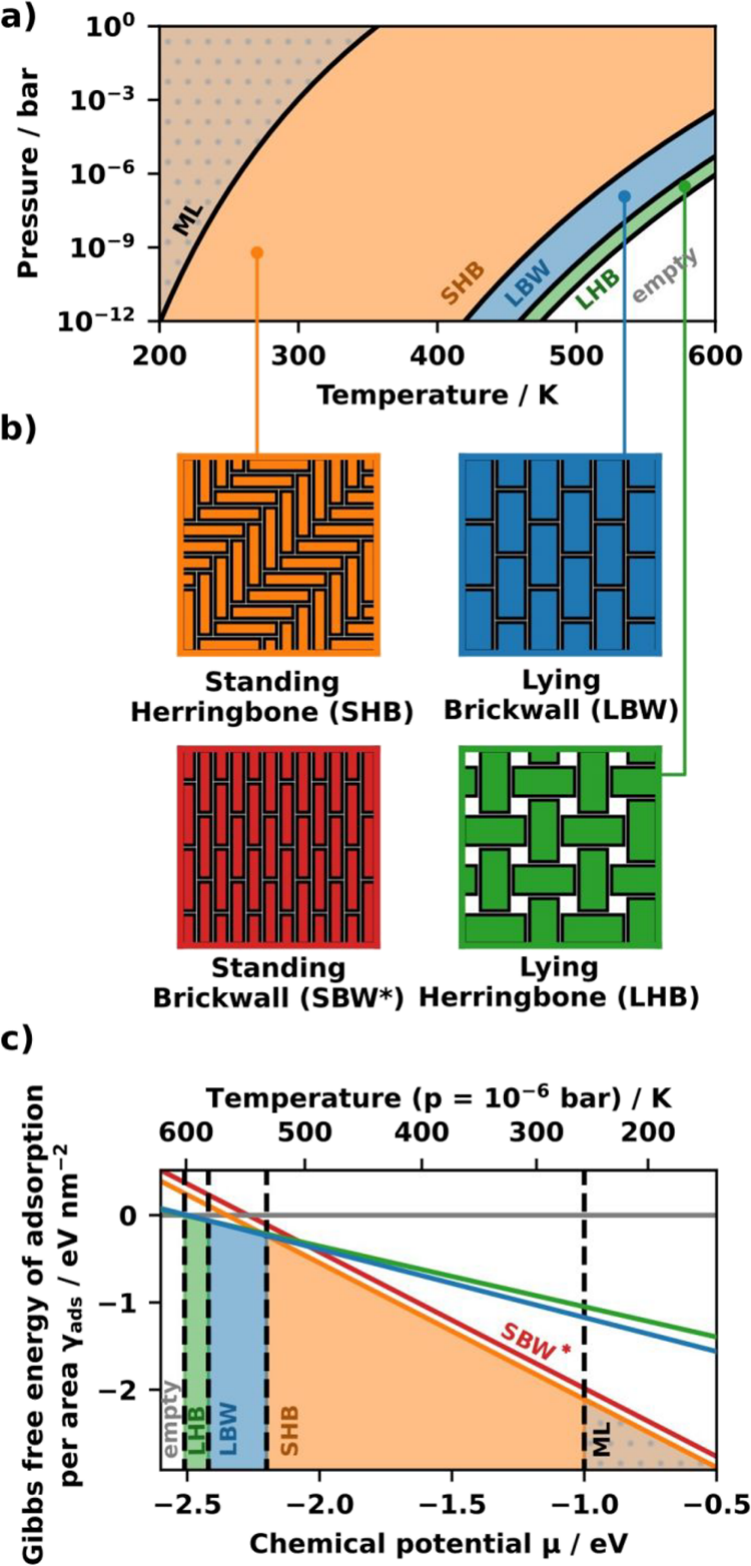
a) Thermodynamic phase diagram of the monolayer structures of the model system based on Equation 1. The same color coding as in Figure 1 is used. The dotted area is the multilayer region (ML) b) The four surface structures of the model. c) The Gibbs free energy of adsorption per area plotted over the chemical potential of the molecular gas reservoir. The filled area corresponds to the thermodynamically stable structure at this chemical potential range.

Three of the monolayer structures (LBW, LHB, and SHB) are thermodynamically stable, as shown in the *p*(*T*) and γ_ads_(μ) thermodynamic phase diagrams in Figure 3. At room temperature, the phase diagram shows that the standing herringbone structure (SHB) is thermodynamically stable. The two lying phases, brick-wall (LBW) and herringbone (LHB), are stable at higher temperatures. The standing brickwall structure (SBW*) is only kinetically accessible, making it a metastable phase (indicated by the asterisk symbol) due to its higher energy and identical coverage to the SHB phase. Putting the phases into context with the introduced growth model, the metastable SBW* phase is our target structure (T in Figure 1). The geometrically similar LBW phase acts as the connector polymorph (C), which becomes thermodynamically accessible at higher temperatures, while the SHB phase (S) is the thermodynamically stable phase under ambient conditions.

By leveraging these system properties, we demonstrate that the metastable SBW* structure can be kinetically accessed in kinetic Monte Carlo (kMC) growth simulations at conditions that are experimentally feasible. In total we conduct 100 kMC growth simulations on a periodically repeated 96 Å x 96 Å area realized by a 24 × 24 square grid with a lattice side length of 4 Å. We start the simulations on an initially empty surface at a temperature of 350 K and a molecular partial pressure of 10^–6^ bar. The slightly elevated temperature facilitates the formation of the thermodynamically stable phase, while still being at the low end of typical hot-wall epitaxy deposition procedures.^13^ Under these conditions the standing herringbone (SHB) phase is thermodynamically stable and emerges on the surface. As expected from Ostwald’s rule, the low coverage phase of lying molecules in the LHB pattern builds up first within one second, as shown in Figure 4a. Subsequently the transition to the thermodynamic stable SHB structure occurs, which is completed within several minutes. In principle this step is not needed for the formation of the metastable structure. We could have directly grown the LBW connector phase by starting at a high temperature where this structure is stable, but we want to demonstrate that without any changes in the deposition conditions the metastable SBW* structure is not accessible, and the system always ends up in the thermodynamic minimum.

**Figure 4:**
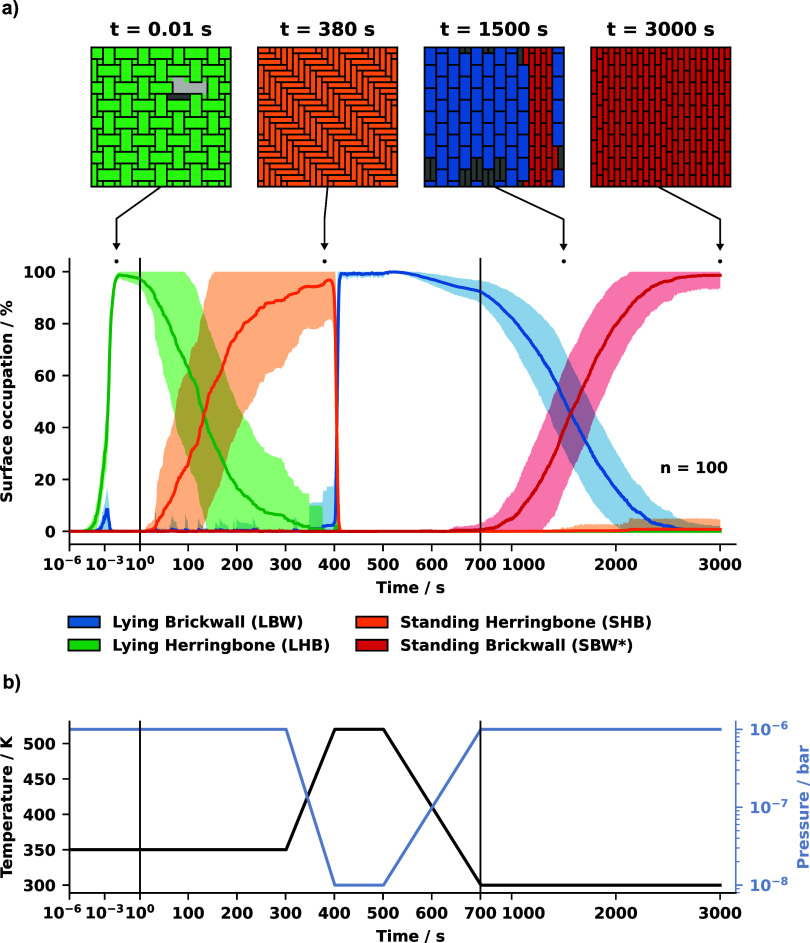
a) Surface occupation of the different surface structures averaged over 100 kMC runs on a 24 × 24 lattice. The shaded area of the curves represents the standard deviation calculated at each time point. The first second of the simulation is plotted in logarithmic scale to display the fast growth of the LHB phase. After that, a linear time axis is used with a different scale after *t* = 700 *s*. Exemplary, the surface configurations of one of the 100 runs at four representative points in time is plotted on top. b) The temperature and pressure protocol during the simulation.

Five minutes (*t* = 300 *s*) after the deposition starts, we change the chemical potential of the gas phase reservoir to a region where the LBW connector phase becomes stable. This is achieved by a linear temperature increase up to 520 K and a logarithmic pressure drop to 10^–8^ bar, similar to a postdeposition thermal treatment.^6^ To keep the simulations realistic, we assume that the temperature change from 350 to 520 K and the pressure drop from 10^–6^ to 10^–8^ bar is not instantaneous, but commences within a duration of 100 s, as shown in Figure 4b. At *t* = 400 *s* the system undergoes a quite sharp phase transition to the stable LBW connector phase, covering close to 100% of the surface in this structure. As the last step, at *t* = 500 *s*, the chemical potential is changed to a region where the standing structure is favored again, which is achieved by cooling the system down to ambient temperature (300 K) and increasing the partial pressure of the adsorbate to 10^–6^ bar. In contrast to the deposition phase during the beginning of the simulation, the system now transforms into the metastable SBW* polymorph. At *t* = 3000 *s*, on average around 99% of the surface is covered with our target phase (see red line in Figure 4a).

Having created the metastable phase, a pressing question for potential technological applications arises: How long does it remain stable? Since the SBW* phase consists of only upright standing molecules only a desorption process of a standing molecule can trigger the degradation of this structure. Exemplarily, at a temperature of 300 K the expected elapsed time for the next desorption process to happen is on average 25·10^6^ years (see Supporting Information). In practice this can be seen as a stable structure.

## Conclusion

In conclusion, we proposed a growth mechanism that allows the targeted formation of metastable surface structures for inorganic/organic interfaces accommodating adsorption geometries in lying and standing molecules, that exhibit a special connector structure with two main properties: First, its Gibbs free energy curve needs to intersect with the Gibbs free energy curve of the metastable target structure and, second, the transition process from the connector phase to this structure needs to be preferred compared to the transition back to the thermodynamic stable structure. For a proof of concept, we conducted kinetic Monte Carlo growth simulations of a simplified interface model of rectangular molecules. The results showed that after applying a specific temperature and pressure protocol to the system a significant area of the simulation cell is occupied with the anticipated metastable structure.

## Supplementary Material



## Data Availability

All the data used in this work and the adapted kMC time increment algorithm code for time-varying process rates are available from the authors upon reasonable request.

## Notes

Discussion of the Gibbs-free energy calculation of monolayers; derivation of the Bronsted-Evans–Polanyi principle of the investigated system; additional computational details of the kMC simulations and the surface composition evaluation; detailed description of the developed algorithm for time-dependent process rates in kMC (PDF)
