# Supplementary material for: Metastable Monolayer Formation through a Connector Structure
Source: J Phys Chem C Nanomater Interfaces. 2025 Jul 9;129(28):13023–9. doi: 10.1021/acs.jpcc.5c02249 (PMC12278309; doi:10.1021/acs.jpcc.5c02249)
Supplement: Supplementary file 1 [file jp5c02249_si_001.pdf]

Supporting Information for ”Metastable Monolayer Formation through a Connector Structure”

Simon B. Hollweger, Anna Werkovits, Oliver T. Hofmann

Institute of Solid State Physics, TU Graz, Petersgasse 16/II, 8010 Graz, Austria

Table of Contents

S1. Gibbs free energy and chemical potential calculation 1
S2. Time varying rates in kinetic Monte Carlo 4
S3. Kinetic Monte Carlo simulation setup 8
S4. Surface composition evaluation 11
S5. Lifetime estimation 13
S6. Brønsted-Evans-Polanyi principle 14
S7. References 16

S1. Gibbs free energy and chemical potential calculation

The Gibbs free energy of adsorption per area γ of an structure is given by [1]

$$\gamma = \frac{1}{A}\left(\Delta G(T) - \mu_{\mathrm{mol}}(T,p)N\right) \tag{S1}$$

where ΔG is the Gibbs free energy difference between the clean surface and the surface with adsorbed molecules on it, μ_{mol} the chemical potential of one molecule in the gas reservoir at given temperature T and partial molecular gas pressure p and N is the number of molecules adsorbed in the surface area A. For simplicity we approximated the Gibbs free energy ΔG with

the adsorption energy E^{ads} and ignore the entropic, work and vibrational contributions. The entropic and work contributions are usually small and negligible.[1,2] The vibrational contribution cancels out to a certain degree with the vibrational contribution of the chemical potential, and it is therefore in this conceptual study justified to be neglected in both terms ΔG and μ_{mol}. With all these approximations we end up with the equation introduced in the main text reading

$$\gamma = \frac{1}{A}\left(E^{\text{ads}} - \mu(T,p)N\right) \tag{S2}$$

For calculating the chemical potential μ a 9-10 anthraquinone molecule was used as reference (Figure S1). The chemical potential μ_{mol} was calculated according to the thermodynamical ideal gas laws, as it is common in *Ab-Initio Thermodynamics*, (details can be found in Rogal and Reuter[1]). The chemical potential consists of several contributions[3], that read

$$\mu_{\text{mol}} = \mu^{\text{trans}} + \mu^{\text{rot}} + \mu^{\text{elec}} + \mu^{\text{vib}} + \mu^{\text{nucl}}. \tag{S3}$$

As discussed in the work of Rogal and Reuter[1] the contribution μ^{nucl} from the nuclei is negligibly small. The vibrational part μ^{vib} is also neglected for reasons already mentioned above. The electronic contribution is given by

$$\mu^{\text{elec}} = E^{\text{tot}}_{\text{mol}} - k_B T \ln(I^{\text{spin}}) \tag{S4}$$

where the total electron energy $E^{\text{tot}}_{\text{mol}}$ is set to zero as it just introduces an offset. The contribution from the spin degeneracy I^{spin} is zero for our reference molecule. With this, the used approximated chemical potential μ for a nonlinear molecule is then given by[3,4]

$$\mu(T,p) = \mu^{\text{trans}} + \mu^{\text{rot}} \tag{S5}$$

with

$$\mu^{\text{trans}} = -k_B T \ln\left[\left(\frac{2\pi m}{h^2}\right)^{\frac{3}{2}} \frac{(k_B T)^{\frac{5}{2}}}{p}\right] \tag{S6}$$

and

$$\mu^{\text{rot}} = -k_B T \ln\left[\frac{\sqrt{\pi I_1 I_2 I_3}}{\sigma}\left(\frac{8\pi^2 k_B T}{h^2}\right)^{\frac{3}{2}}\right]. \tag{S7}$$

All the used molecular constants of Anthraquinone are listed in Table S1.

Table S1. Used parameters for chemical potential calculation

Parameter	Symbol	Value
**Mass**	$\boldsymbol{m}$	$3.458 \cdot 10^{-25}$ kg
**Moments of inertia**	$\boldsymbol{I_1}$	$7.636 \cdot 10^{-45}$ kg m²
	$\boldsymbol{I_2}$	$1.895 \cdot 10^{-44}$ kg m²
	$\boldsymbol{I_3}$	$2.659 \cdot 10^{-44}$ kg m²
**Symmetry number**	$\boldsymbol{\sigma}$	4

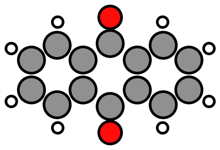

Figure S1. The used 9-10 anthraquinone reference molecule for calculating the chemical potential. The carbon atoms are colored grey, the hydrogens white and the oxygen red.

S2. Time varying rates in kinetic Monte Carlo

The simulation time in a kinetic Monte Carlo run is propagated according to a zeroth order Poisson process.[5,6] This means we draw the random time step Δt from an exponential probability distribution given by

$$\Delta t \sim p(\Delta t | k_{\mathrm{tot}}) = k_{\mathrm{tot}} e^{-k_{\mathrm{tot}} \Delta t} \tag{S8}$$

where k_{tot} is the total sum of all process rates in the kMC system at the current kMC step. We can sample this distribution with a uniformly distributed random number $r \sim \mathcal{U}(0,1)$ by using

$$\Delta t = -\frac{\ln(1-r)}{k_{\mathrm{tot}}} \tag{S9}$$

This probability distribution is only valid for constant rates k_{tot}. However, in this study we have to consider time varying rates in the system and we need to determine the time step Δt from a distribution which considers the time dependence. The probability distribution for the general time dependent case at time point t is given by [6,7]

$$\Delta t \sim p(\Delta t | t, k_{\mathrm{tot}}) = k_{\mathrm{tot}}(t + \Delta t) e^{-\int_0^{\Delta t} k_{\mathrm{tot}}(t+\Delta t')d\Delta t'}. \tag{S10}$$

To sample this time dependent distribution with an uniformly distributed random variable $r \sim \mathcal{U}(0,1)$ we first need to calculate the cumulative distribution function

$$F_c(\Delta t | t, k_{\mathrm{tot}}) = \int_0^{\Delta t} p\,(\Delta t' | t, k_{\mathrm{tot}}) d\Delta t' = \\ = \int_0^{\Delta t} k_{\mathrm{tot}}\,(t + \Delta t') e^{-\int_0^{\Delta t'} k_{\mathrm{tot}}(t+\Delta t'')d\Delta t''} d\Delta t' \tag{S11}$$

With the substitution $\mu = \int_0^{\Delta t'} k_{\mathrm{tot}}\,(t + \Delta t'')d\Delta t''$ and $d\mu = k_{\mathrm{tot}}(t + \Delta t')d\Delta t'$ we find

$$F_c(\Delta t|t, k_{\text{tot}}) = \int_{\mu(0)}^{\mu(\Delta t)} e^{-\mu}\, d\mu =$$

$$= -e^{-\mu}\Big|_{\mu(0)=0}^{\mu(\Delta t)=\int_0^{\Delta t} k_{\text{tot}}(t+\Delta t')d\Delta t'} = \tag{S12}$$

$$= 1 - e^{-\int_0^{\Delta t} k_{\text{tot}}(t+\Delta t\prime)d\Delta t\prime}.$$

With the inverse cumulative distribution function F_c^{-1} and the uniformly distributed $r \sim \mathcal{U}(0,1)$ we can find now the random time step Δt following the time dependent distribution function $p(\Delta t|t, k_{\text{tot}})$ in Equation (S10)

$$\Delta t = F_c^{-1}(r). \tag{S13}$$

Unfortunately, it is not possible to find a closed form for F_c^{-1}. We can only write an implicit equation of the form

$$\ln(1-r) + \int_0^{\Delta t} k_{\text{tot}}\,(t + \Delta t')d\Delta t' = 0 \tag{S14}$$

that needs to be solved for Δt. Notice if $k_{\text{tot}}(t)$ is changing only slowly in the time interval $(t, t + \Delta t)$ we can approximate it with a constant k_{tot} and can use the equation for the time-independent case mentioned in Equation (S9). However, as already described in the main text, there exist cases where the time step Δt is large and this approximation is not justified. To account for this, we adapted the time propagation algorithm in *kmos3* and solved the implicit Equation (S14) on a time grid τ_i that is fine enough to represent the time dependent total rate k_{tot} sufficiently exact with a step function. In case of a step function for k_{tot} the integral in Equation (S14) can be rewritten as a sum over rectangles and can be solved iteratively. If we

assume that the current simulation time t_{kMC} lies within the time grid interval (τ_{i-1}, τ_i), we can write for the integral in Equation (S14)

$$\int_0^{\Delta t} k_{tot}(t_{kMC} + \Delta t')d\Delta t' \approx$$

$$\approx \begin{cases} k_{\mathrm{tot}}(\tau_{i-1})\Delta t & \text{for } t_{\mathrm{kMC}} + \Delta t \leq \tau_i \\ k_{\mathrm{tot}}(\tau_{i-1})[\tau_i - t_{\mathrm{kMC}}] + & \text{for } \tau_i < t_{\mathrm{kMC}} + \Delta t \leq \tau_{i+1} \\ \quad + k_{\mathrm{tot}}(\tau_i)[t_{\mathrm{kMC}} + \Delta t - \tau_i] & \\ \qquad \vdots & \qquad \vdots \\ k_{\mathrm{tot}}(\tau_{i-1})[\tau_i - t_{\mathrm{kMC}}] + & \text{for } \tau_{i+N} < t_{\mathrm{kMC}} + \Delta t \leq \tau_{i+N+1} \\ \quad + [\sum_{k=1}^{N} k_{\mathrm{tot}}(\tau_{i+k-1})[\tau_{i+k} - \tau_{i+k-1}]] & \\ \quad + k_{\mathrm{tot}}(\tau_{i+N})[t_{\mathrm{kMC}} + \Delta t - \tau_{i+N}] & \end{cases} . \tag{S15}$$

In Figure S2 a sketch for the approximation of the integral in Equation (S15) is shown.

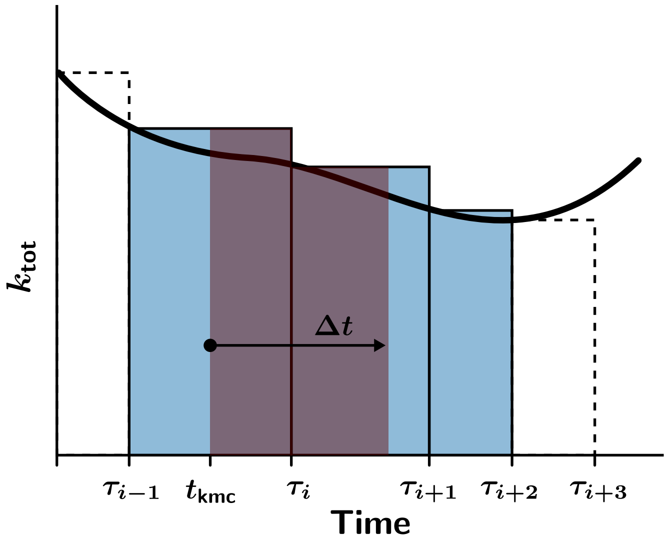

Figure S2. Sketch for determining the approximation of the integral from Equation (S15) on a time grid.

With this approximation at hand, we can formulate an algorithm that gives us the time step Δt that solves Equation (S14). We simply have to insert the individual cases from Equation (S15) into (S14) starting from the top and solve for Δt. We can stop this iterative scheme as soon as a found time step Δt fulfills the case condition or the final simulation time t_{final} is exceeded. In practice the following steps are required:

1. Compute the time step corresponding to the first case in (S15) that gives

$$\Delta t = -\frac{\ln(1-r)}{k_{\mathrm{tot}}(\tau_{i-1})} \tag{S16}$$

2. Then check if $t_{\mathrm{kMC}} + \Delta t \leq \tau_i$, if yes, we stop and can propagate the simulation time with Δt. This is just a regular kMC time step as it would be the case for the time independent case.
3. If the determined Δt in step 1 overshoots the current time interval we initialize a counter $N = 0$ and propose a new time step with

$$\begin{aligned} \Delta t &= \tau_{i+N} - t_{\mathrm{kMC}} + \\ &+ \frac{-\ln(1-r) - k_{\mathrm{tot}}(\tau_{i-1})[\tau_i - t_{\mathrm{kMC}}] - \sum_{k=1}^{N} k_{\mathrm{tot}}\,(\tau_{i+k-1})[\tau_{i+k} - \tau_{i+k-1}]}{k_{\mathrm{tot}}(\tau_{i+N})} \end{aligned} \tag{S17}$$

4. Now we need to check again if $t_{\mathrm{kMC}} + \Delta t \leq \tau_{i+N+1}$. If this is fulfilled, we are done and found a proper time step. If it is not fulfilled, we increment the counter ($N = N + 1$) and repeat this step until it is fulfilled. Notice that in the first iteration with $N = 0$ the sum term in Equation (S17) is empty and therefore zero. We also terminate the time step search if we exceed the final simulation time t_{final}.

In Figure S3 a flowchart of this algorithm for determining Δt can be found.

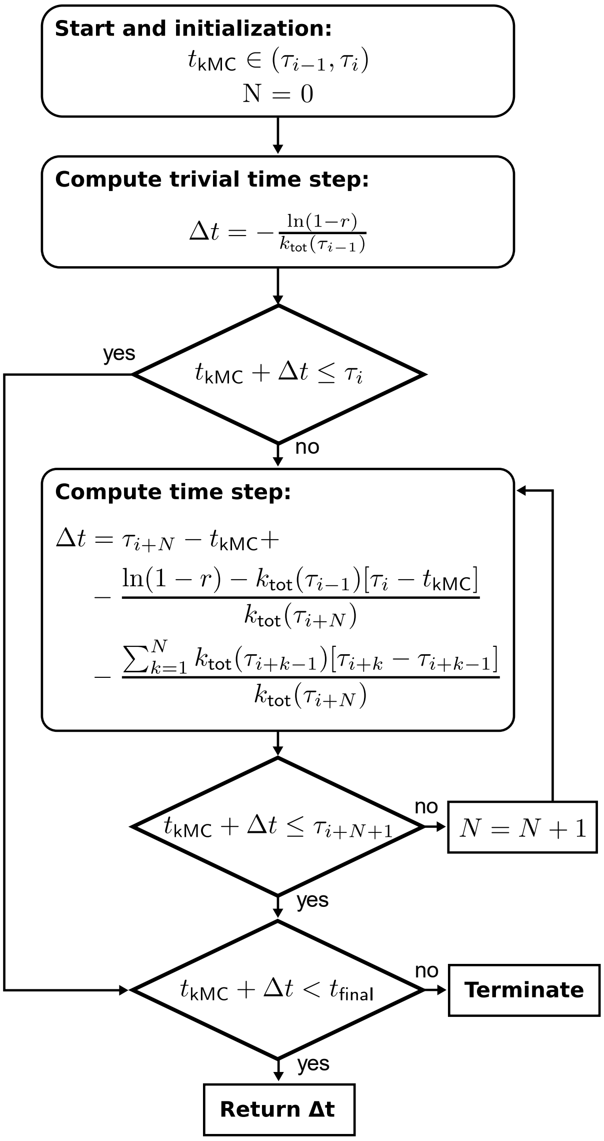

Figure S3. Algorithm for determining the kMC time step in the case of time varying rate constants

With that algorithm we can propagate the simulation time in our model even for large time jumps that are in the range of the variation times of the rate constants.

S3. Kinetic Monte Carlo simulation setup

The kinetic Monte Carlo simulations were performed with an adapted version of the *kmos3* simulation package.[8] The kMC models were generated with the on-the-fly (otf) backend that considers the intermolecular interactions during runtime. For the interactions only the nearest

neighbors were considered, as it can be seen in Figure 2b in the main text. Additionally, the previously described algorithm for determining the time step in the case of time dependent rate constants was implemented in *kmos3*[9] by us and used in all simulation runs. All the parameters used for the time acceleration scheme[10] implemented in *kmos3* are listed in Table S2.

Table S2. Used parameters for the time acceleration scheme. The names are the ones used in the *kmos3* code documentation[9].

Parameter	Value
**buffer_parameter**	500
**sampling_steps**	20
**execution_steps**	200
**threshold_parameter**	0.2

All possible elementary processes except the adsorption and desorption processes are visualized in Figure S4. The used energy barriers needed for evaluating the Arrhenius equation are listed in table Table S3. All attempt frequencies for all elementary processes were set to $f = 10^{12}\ \mathrm{s}^{-1}$.

Table S3. All barriers used for calculating the on-surface processes visualized in Figure S4. The corresponding attempt frequencies are all equal and set to $f = 10^{12}\ \mathrm{s}^{-1}$.

Parameter	Value
**Lying diffusion barrier $\Delta E_{\mathrm{L}}^{\mathrm{diff}}$**	0.3 eV
**Standing diffusion barrier $\Delta E_{\mathrm{S}}^{\mathrm{diff}}$**	0.3 eV
**Stand up barrier $\Delta E_{\mathrm{LS}}^{\mathrm{reor}}$**	0.45 eV
**Fall-over barrier $\Delta E_{\mathrm{SL}}^{\mathrm{reor}}$**	0.25 eV
**Lying rotation barrier $\Delta E_{\mathrm{L}}^{\mathrm{rot}}$**	0.3 eV
**Standing rotation barrier $\Delta E_{\mathrm{S}}^{\mathrm{rot}}$**	0.3 eV

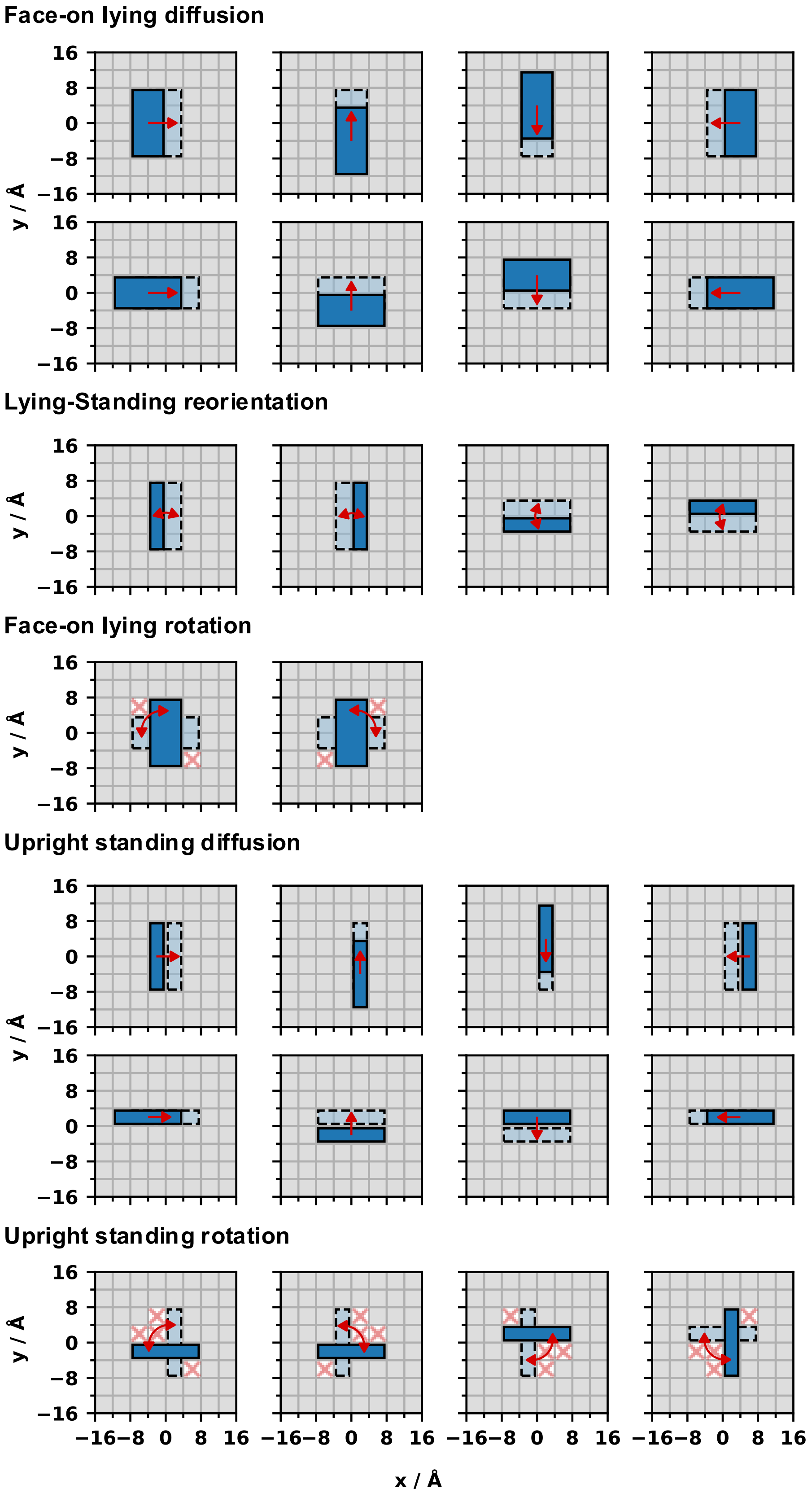

Figure S4. All possible elementary processes and the corresponding requirements for empty sites for the process to be possible.

S4. Surface composition evaluation

For evaluating the areal occupation of the different polymorphs on the surface during a kMC run we use a simple expansion algorithm.

1. The first step is to iterate through the surface sites and check if one can find a molecular neighborhood that is identical to a unit cell of the structures one is checking for.

2. If a unit cell is found, we try to expand it by checking for neighbor molecules that obey the periodicity of the found unit cell. If a neighbor molecule fits in the structure, the search is continued from this molecule onwards. The expansion of this domain stops if no neighbors can be found anymore.

3. As soon as a domain expansion is completed one checks if it satisfies a minimum size requirement to be considered as domain (e.g. number of domain molecules $n > n_{\min}$). Then we go back to step 1 and further iterate the lattice sites to search for the next unit cell to be expanded. All molecules that are already categorized are skipped.

When every lattice site and unit cell type is checked we end up with a list of domains that can be summed up to give the areal occupations of the different structures. The minimum size to be considered as a domain of a structure, $n_{\min} = 4$ was used for lying structures and $n_{\min} = 6$ for standing structures.

In the histogram in Figure S5 the final yield (at $t = 3000$ s) of the target SBW* structure of all 100 performed kMC simulations is shown. The average yield is around 99 %.

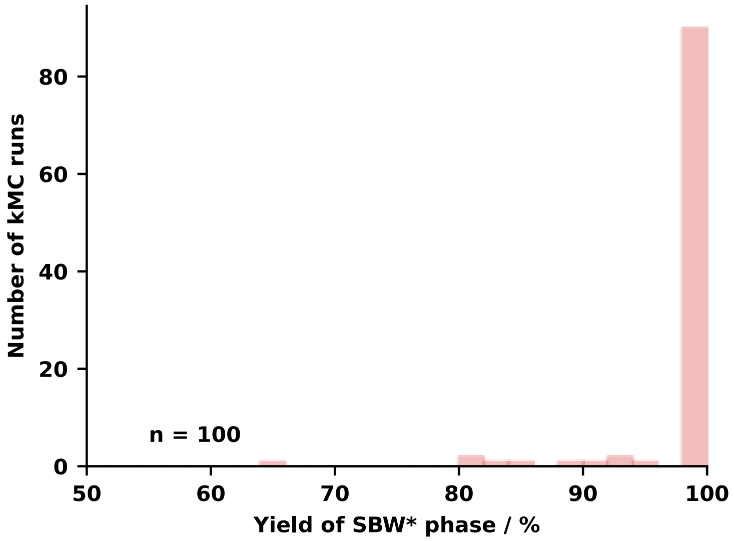

Figure S5. Histogram of the number of kMC simulations binned by the yield of the metastable SBW* phase after $t = 3000$ s.

In Figure S6 the time evolution of the mean Gibbs free energy of adsorption of the system is visualized with the theoretically calculated Gibbs free energies of adsorption of the pure surface structures in the background.

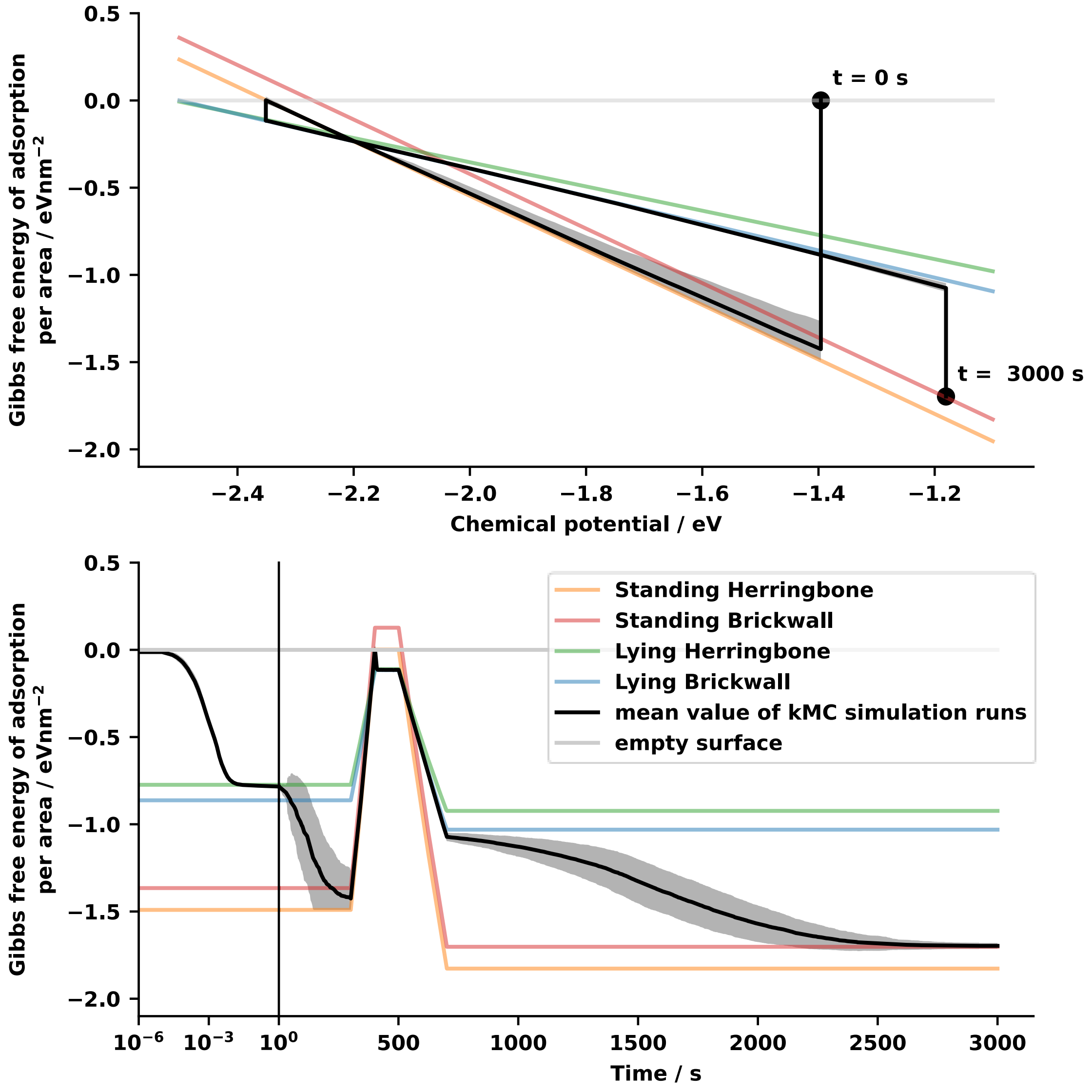

Figure S6. Time evolution of the mean Gibbs free energy of adsorption per area of all 100 runs: (a) the energy trajectory plotted over the chemical potential of the molecular gas reservoir; (b) the energy trajectory plotted over the simulation time

S5. Lifetime estimation

To estimate the stability of the SBW* phase it is sufficient to focus on the rate constant of a standing molecule within the SBW* lattice desorbing from the surface. This type of process is the only available process in the situation of a fully closed standing monolayer and therefore the only one capable of triggering a phase transition to the thermodynamic stable SHB phase. The average waiting time $\langle t_{\mathrm{des}} \rangle$ until the next desorption process is happening can be estimated

by determining the expectation value of the underlying Poisson distribution of this process and is given by $\langle t_{\mathrm{des}} \rangle = 1/k_{\mathrm{des}}$. The desorption rate k_{des} is given by Equation (6) of the main text. At a temperature of $T = 300$ K (pressure p is not relevant as it cancels between k_{ads} and μ^{trans} in Equation (6)) we find

$$\langle t_{\mathrm{des}} \rangle = 7.93 \times 10^{14}\ \mathrm{s} = 25.15 \times 10^{6}\ \mathrm{years}. \tag{S18}$$

This sufficiently shows the relative stability of the metastable SBW* phase.

S6. Brønsted-Evans-Polanyi principle

In general, the Bronsted-Evans-Polanyi[11–16] (BEP) principle states that the activation energy barrier ΔE and the enthalpy difference ΔH between initial and final state of reactions of similar type are in a linear relation. This is described by a proportionality parameter $\alpha \in [0,1]$ and a reference energy $E_{\ddagger}^{R}$.

$$\Delta E = \alpha \Delta H + E_{\ddagger}^{R} \tag{S19}$$

The parameters α and $E_{\ddagger}^{R}$ are usually determined by performing a linear fit on calculated transition energy barriers of different processes of the same reaction family (e.g. only diffusion processes).

For the kinetic Monte Carlo simulation, we need to know how the energy barrier ΔE of a process changes between the case where interacting neighbor molecules are present and the non-interacting single molecule case. We can use the Bronsted-Evans-Polanyi relation in Equation (S19) to write for the interacting transition barrier $\Delta E'$ and the non-interacting case ΔE^{0}

$$\Delta E^{0} = \alpha \underbrace{\left(E_{\text{fin}}^{0} - E_{\text{ini}}^{0}\right)}_{\Delta H^{0}} + E_{\ddagger}^{R} \tag{S20}$$

$$\Delta E' = \alpha \underbrace{\left(E'_{\text{fin}} - E'_{\text{ini}}\right)}_{\Delta H'} + E_{\ddagger}^{R} \tag{S21}$$

where E_{ini} and E_{fin} are the initial and final energies of the molecule, see Figure S7. The change in the energy barrier from the non-interacting case to the interacting case is denoted by $\Delta\Delta E^{\text{int}}$ and can be calculated by the difference between these two expressions above

$$\begin{aligned} \Delta\Delta E^{\text{int}} &= \Delta E' - \Delta E^{0} = \alpha[(E'_{\text{fin}} - E'_{\text{ini}}) - (E_{\text{fin}}^{0} - E_{\text{ini}}^{0})] = \\ &= \alpha\left[\underbrace{\left(E_{\text{fin}}^{0} + E_{\text{fin}}^{\text{int}}\right)}_{E'_{\text{fin}}} - \underbrace{\left(E_{\text{ini}}^{0} + E_{\text{ini}}^{\text{int}}\right)}_{E'_{\text{ini}}} - \left(E_{\text{fin}}^{0} - E_{\text{ini}}^{0}\right)\right] = \\ &= \alpha[E_{\text{fin}}^{\text{int}} - E_{\text{ini}}^{\text{int}}]. \end{aligned} \tag{S22}$$

We found now the expression from the main text (Equation (8)) for correcting the non-interacting energy barrier ΔE^{0} to find the interacting energy barrier

$$\Delta E' = \Delta E^{0} + \Delta\Delta E^{\text{int}}. \tag{S23}$$

We can see that only the interaction energies $E_{\text{fin}}^{\text{int}}$ and $E_{\text{ini}}^{\text{int}}$ of the molecules in the initial and final state are necessary to account for the effect of the interactions on the transition barrier.

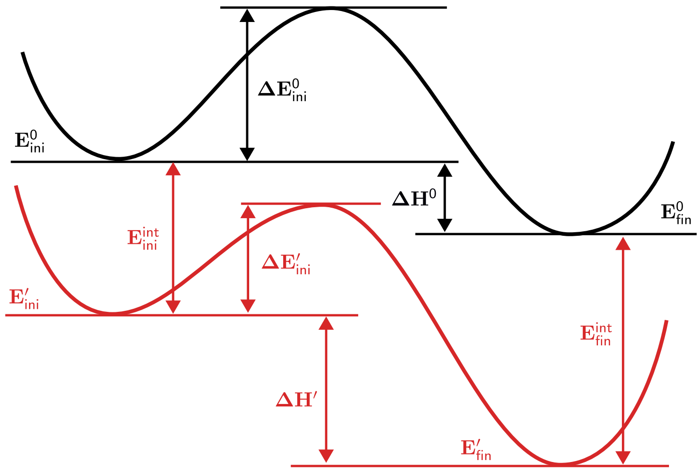

Figure S7. Reaction path of a single molecule on the surface with no interactions (black line) and the same process but with attractive neighbor interactions at initial and final state (red line).

These corrections are used in all the performed kinetic Monte Carlo simulations. For the reorientation processes from a face-on lying geometry to a long-edge standing orientation a scaling value of $\alpha = 0.7$ was used, for all other processes α was set to 0.5 .

S7. References

(1) Rogal, J.; Reuter, K. Ab Initio Atomistic Thermodynamics for Surfaces: A Primer; 2007.
(2) Wachter, C.; Werkovits, A.; Hofmann, O. T. Phase Diagrams for Organic/Metal Interfaces: Significance of Configurational and Vibrational Energies. *J. Chem. Phys.* **2025**, *162* (20), 204705. https://doi.org/10.1063/5.0252855.
(3) Cramer, C. J. *Essentials of Computational Chemistry: Theories and Models*; John Wiley & Sons, 2013.
(4) McQuarrie, D. A. *Statistical Mechanics*; University Science Books, 2000.
(5) Andersen, M.; Panosetti, C.; Reuter, K. A Practical Guide to Surface Kinetic Monte Carlo Simulations. *Front. Chem.* **2019**, *7*.
(6) Pineda, M.; Stamatakis, M. Kinetic Monte Carlo Simulations for Heterogeneous Catalysis: Fundamentals, Current Status, and Challenges. *J. Chem. Phys.* **2022**, *156* (12), 120902. https://doi.org/10.1063/5.0083251.
(7) Gardiner, C. W. *Handbook of Stochastic Methods for Physics, Chemistry, and the Natural Sciences*; Springer-Verlag, 1985.

(8) Hoffmann, M. J.; Matera, S.; Reuter, K. Kmos: A Lattice Kinetic Monte Carlo Framework. *Comput. Phys. Commun.* **2014**, *185* (7), 2138–2150. https://doi.org/10.1016/j.cpc.2014.04.003.
(9) *kmos3.org*. https://kmos3.org/ (accessed 2025-02-18).
(10) Andersen, M.; Plaisance, C. P.; Reuter, K. Assessment of Mean-Field Microkinetic Models for CO Methanation on Stepped Metal Surfaces Using Accelerated Kinetic Monte Carlo. *J. Chem. Phys.* **2017**, *147* (15), 152705. https://doi.org/10.1063/1.4989511.
(11) Evans, M. G.; Polanyi, M. Some Applications of the Transition State Method to the Calculation of Reaction Velocities, Especially in Solution. *Trans. Faraday Soc.* **1935**, *31* (0), 875–894. https://doi.org/10.1039/TF9353100875.
(12) Evans, M. G.; Polanyi, M. Further Considerations on the Thermodynamics of Chemical Equilibria and Reaction Rates. *Trans. Faraday Soc.* **1936**, *32* (0), 1333–1360. https://doi.org/10.1039/TF9363201333.
(13) Evans, M. G.; Polanyi, M. Inertia and Driving Force of Chemical Reactions. *Trans. Faraday Soc.* **1938**, *34* (0), 11–24. https://doi.org/10.1039/TF9383400011.
(14) Bell, R. P. The Theory of Reactions Involving Proton Transfers. *Proc. R. Soc. Lond. Ser. A* **1936**, *154*, 414–429. https://doi.org/10.1098/rspa.1936.0060.
(15) Bronsted, J. N. Acid and Basic Catalysis. *Chem. Rev.* **1928**, *5* (3), 231–338. https://doi.org/10.1021/cr60019a001.
(16) Brönsted, J. N.; Pedersen, K. Die katalytische Zersetzung des Nitramids und ihre physikalisch-chemische Bedeutung. *Z. Für Phys. Chem.* **1924**, *108U* (1), 185–235. https://doi.org/10.1515/zpch-1924-10814.
